# Coiled-Coil Domain Containing 80 Suppresses Nonylphenol-Induced Colorectal Cancer Cell Proliferation by Inhibiting the Activation of ERK1/2

**DOI:** 10.3389/fcell.2021.759820

**Published:** 2021-10-22

**Authors:** Jing Wang, Yuan-wei Zhang, Nian-jie Zhang, Shuo Yin, Du-ji Ruan, Nian He, Xu Chen, Xue-feng Yang

**Affiliations:** ^1^School of Life Sciences and Technology, Wuhan University of Bioengineering, Wuhan, China; ^2^Department of Gastrointestinal Surgery, The Second Affiliated Hospital of Zunyi Medical University, Zunyi, China

**Keywords:** nonylphenol, colorectal cancer, CCDC80, ERK1/2, cell proliferation

## Abstract

Recently, the effect of endocrine-disrupting chemicals on the cancer procession has been a concern. Nonylphenol (NP) is a common environmental estrogen that has been shown to enhance the proliferation of colorectal cancer (CRC) cells in our previous studies; however, the underlying mechanism remains unclear. In this study, we confirmed the increased concentration of NP in the serum of patients with CRC. RNA sequencing was used to explore the differentially expressed genes after NP exposure. We found 16 upregulated genes and 12 downregulated genes in COLO205 cells after NP treatment. Among these differentially expressed genes, we found that coiled-coil domain containing 80 (CCDC80) was downregulated by NP treatment and was associated with CRC progression. Further experiments revealed that the overexpression of CCDC80 significantly suppressed NP-induced cell proliferation and recovered the reduced cell apoptosis. Meanwhile, the overexpression of CCDC80 significantly inhibited the activation of ERK1/2 induced by NP treatment. ERK1/2 inhibitor (PD98059) treatment also suppressed NP-induced CRC cell growth, but the overexpression of CCDC80 did not enhance the effect of ERK1/2 inhibitor. Taken together, NP treatment significantly inhibited the expression of CCDC80, and the overexpression of CCDC80 suppressed NP-induced CRC cell growth by inhibiting the activation of ERK1/2. These results suggest that NP could induce CRC cell growth by influencing the expression of multiple genes. CCDC80 and ERK1/2 inhibitors may be suitable therapeutic targets in NP-related CRC progression.

## Introduction

Colorectal cancer (CRC) is the third most commonly diagnosed cancer. It was also the second leading cause of cancer-related deaths worldwide in 2018 (Bray et al., 2018). Apart from genetic factors, the main risk factors for CRC are environmental risk factors such as obesity, alcohol consumption, physical inactivity, unhealthy diets, and smoking (Keum and Giovannucci, 2019). In recent years, the relationship between environmental pollution and carcinogens has become a concern.

Endocrine disrupting chemicals (EDCs), also known as environmental hormones, are exogenous chemicals existing in the environment that can interfere with the endocrine system of humans or animals. Many studies have demonstrated that EDCs are involved in the occurrence and development of cancers (Marotta et al., 2019; Noorimotlagh et al., 2020; Li et al., 2021). Xenoestrogens are a class of EDCs that have estrogenic activity and can be found in plastics, detergents, surfactants, pesticides, and industrial chemicals (Chighizola and Meroni, 2012). Nonylphenol (NP) is mainly used in the production of surfactants, as well as in antioxidants, textile printing and dyeing auxiliaries, lubricating oil additives, pesticide emulsifiers, resin modifiers, resin and rubber stabilizers, and other fields (Noorimotlagh et al., 2020). For decades, NPs have been discharged into the environment through wastewater, petrochemical products, industrial fumes, even ubiquitous in food (Guenther et al., 2002; Raecker et al., 2011; Acir and Guenther, 2018). Furthermore, NP is persistent and bio-accumulative; thus, it can be scaled up through the food chain (Rivero et al., 2008). Previous studies have demonstrated that NP could induce female-related cancers, such as breast and ovarian cancers, by binding to nuclear receptors (In et al., 2015; Kim et al., 2015). Recently, the relationship between NP exposure and risk of CRC progression has been a concern. The risk of CRC has significantly increased in populations living near the industries, which release NP, in Spain (Garcia-Perez et al., 2020). Our previous studies have revealed that NP promoted CRC cell proliferation (Xie et al., 2019; Yang et al., 2019). However, the mechanism of action of NP on CRC progression requires further studies.

In the present study, we performed transcriptome sequencing to explore the transcriptome changes in CRC cells after NP treatment. We found that the coiled-coil domain containing 80 (CCDC80), a tumor suppressor in CRC, was significantly reduced after NP exposure (Grill et al., 2018). The results of the current study reveal the underlying mechanism of NP exposure in CRC progression and provide novel insights into CRC occurrence and progression.

## Materials and Methods

### Concentration of Nonylphenol in Serum

The study participants were divided into a CRC group and healthy group (control group). With respect to the CRC group, all patients were hospitalized in the gastrointestinal surgery department of the Second Affiliated Hospital of Zunyi Medical University from January 2020 to January 2021. A total of 143 patients, ranging from 21 to 84 years old, with an average age of 62.01 years old, were diagnosed with CRC *via* colonoscopy and pathology testing. The 143 patients comprised 89 males, ranging from 21 to 84 years old, with an average age of 60.28 years and 54 females, ranging from 32 to 76 years, with an average age of 64.74 years. For the control group, a total of 143 healthy subjects were selected after physical examination at our hospital during the same period. The participants’ ages ranged from 22 to 78 years, with an average age of 61.87 years. The control group comprised 89 males, aged between 22 and 77 years, with an average age of 61.2 years, and 54 females, ranging from 30 to 78 years old, with an average age of 63.57 years. There were no significant differences in age or sex between the two groups (*P* > 0.05). The serum from these subjects was stored at −80°C. All experimental procedures were carried out in accordance with the Chinese legislation regarding medical ethics and were approved by the Medical Ethics Committee of Zunyi Medical University [(2019) H-008].

To test the concentration of serum NP, a 0.5 mL serum sample was mixed with 4 mL of n-hexane-ether extract solution (volume ratio of 7:3), after which the mixture was vortexed for 30 s and allowed to stand for 15 min. The supernatant was then dried in a 50°C water bath. The samples were dissolved in 0.5 mL acetonitrile and detected by high performance liquid chromatography (HPLC).

### Cell Culture and Treatment

COLO205, SW480, and HCT116 cells were obtained from iCell (Shanghai, China). COLO205 cells were cultured in RPMI-1640 medium supplemented with 10% fetal bovine serum (FBS), HCT116 cells were cultured in dulbecco’s modified eagle medium supplemented with 10% FBS, and SW480 cells were cultured in Leibovitz’s L-15 medium supplemented with 10% FBS. All cells were cultured in an incubator at 37°C with 5% CO_2_. All media and FBS were purchased from Thermo Fisher Scientific (Shanghai, China).

Nonylphenol (N109556, Aladdin, Shanghai, China) was dissolved in Dimethyl sulfoxide (DMSO) to 100 mM and diluted to different concentrations (10^–7^, 10^–6^, and 10^–5^ M) to treat the cells.

The ERK1/2 inhibitor (PD98059, MCE, Shanghai, China, the chemical structure showed in Supplementary Figure 1) was dissolved in DMSO to 10 mM and diluted to 20 μM to treat the cells.

Lentiviruses carrying CCDC80 and a negative control (1 × 10^9^ pfu) were generated by GeneChem (Shanghai, China). Before the experiments, the infected COLO205 and SW480 cells were treated with G418 (800 ng/mL) for 2 weeks.

### 5-Ethynyl-2′-Deoxyuridine Assay

Infected COLO205 and SW480 cells were seeded in 24-well plates at a density of 1 × 10^5^ cells/well for 24 h. After treatment with NP (10^–6^ M) or DMSO (*n* = 3 per group) for 24 h, the cells were treated with 5-ethynyl-2′-deoxyuridine (EdU) and counted using the BeyoClickTMEdU-488 cell proliferation kit (Beyotime, Hangzhou, China) according to the manufacturer’s instructions. The cell nuclei were stained with 4′,6-diamidino-2-phenylindole. They were then visualized under a fluorescence microscope, photographed, and the number of positive cells (green fluorescence) were analyzed using the ImageJ software.

### Cell Apoptosis

Cell apoptosis was analyzed using flow cytometry. Cells were seeded into 6-well plates at a density of 5 × 10^5^ cells/well for 24 h and treated with NP (10^–6^ M) for 24 h. Cells were then collected and stained using the Annexin V-FITC/PI cell apoptosis kit (Jiancheng, Nanjing, China).

### Cell Counting Kit-8 Assay

Infected COLO205 and SW480 cells were seeded in 96-well plates at a density of 1 × 10^4^ cells/well for 24 h. After treatment with NP (10^–6^ M) or DMSO (*n* = 6 per group) for 24 h, 10 μL of cell counting kit-8 assay (CCK8) reagent (TransGen, Beijing, China) was added to the cells, after which they were incubated for 4 h in an incubator at 37°C with 5% CO_2_. Absorbance was measured at 450 nm using a microplate analyzer.

### RNA Sequencing

COLO205 cells were treated with NP (10^–6^ M) for 24 h, and then digested with trypsin. The samples were sent to Personalbio (Shanghai, China) for second-generation sequencing. Gene expression was normalized using fragments per kilobase of transcript per million fragments (FPKM). The differentially expressed genes between the control and NP treatment groups were selected by DESeq, with a cutoff of |log2FoldChange| > 1, *P*-value < 0.05. The raw data were uploaded in Gene Expression Omnibus (GEO) as the series number is GSE18222.

### Quantitative PCR

COLO205, SW480, and HCT-116 cells were exposed to NP (10^–7^, 10^–6^, and 10^–5^ M) and collected 24 h after treatment. Total RNA was extracted using TRIzol reagent (Invitrogen, United States). After reverse transcription, the expression of CCDC80, matrix metallopeptidase 19 (MMP19), and Sulfiredoxin-1 (SRXN1) was tested using the QuantStudio^TM^ 6 Flex Real-Time PCR System (Applied Biosystems) using the YBR Green PCR kit (KAPA Biosystems, United States). The relative expression of these genes was analyzed using 2^–ΔΔCt^ method, and glyceraldehyde-3-phosphate dehydrogenase (GAPDH) was used as an internal reference.

### The Cancer Genome Atlas Analysis

The cancer genome atlas (TCGA) analysis of differentially expressed genes after NP treatment was performed using the UALCAN system (Chandrashekar et al., 2017).

### Hematoxylin-Eosin Staining and Immunohistochemistry

Tumor tissue (*n* = 9) and para-cancerous tissue (*n* = 9) were fixed with 4% paraformaldehyde and embedded in paraffin. Immunohistochemistry (IHC) and hematoxylin-eosin (HE) staining were performed after sectioning. The primary antibody used was anti-CCDC80 (1:50, Bioss, Beijing, China). Cells with yellow or brown staining in the nucleus and/or cytoplasm were deemed positive for immunoreactivity. The immunoreactivity was scored as average optical density (IOD) using Image-Pro Plus 6.0 software (IPP6.0).

### Western Blot Assay

The protein expression of treated cells was analyzed using western blotting, as previously described (Kim et al., 2015). The primary antibodies used were anti-GAPDH (Abcam, United Kingdom), anti-ERK1/2, anti-p-ERK1/2, anti-proliferating cell nuclear antigen (PCNA), anti-c-MYC, anti-cyclin D1, anti-Bad, anti-Bcl-2, and anti-Cleaved Caspase-3 (Santa Cruz Biotechnology, United States). The IOD was analyzed using IPP6.0.

### Tumor Xenografts *in vivo*

The mice (BALB/c athymic nude mice, male, 5–6-week-old) information remains the same as previously described (Kim et al., 2015). All experimental procedures were carried out in accordance with the Chinese legislation regarding experimental animals and were approved by the Animal Experiment Ethics Committee of Zunyi Medical University [(2019) A-004]. SW480 cells were digested and resuspended at a concentration of 1.0 × 10^7^ cells/mL. To construct xenograft models, 20 μL of cells were injected into the left posterior flank of each mouse (*n* = 6 per group). Five days after injection, the mice were divided into four groups: (1) control group, (2) CCDC80 group, (3) NP group, and (4) NP + CCDC80 group. Fifty microliters of lentivirus (5 × 10^7^ pfu) carrying CCDC80 or negative control was injected intratumorally every week for 3 weeks (Xie et al., 2019; Park et al., 2020). NP treatment [17 mg/(kg…d)] and other treatments were consistent with our previous study (Kim et al., 2015).

### Statistics

Three parallel experiments were performed for each treatment. Data were analyzed using GraphPad Prism 8 software (GraphPad Software, La Jolla, CA, United States). Data are presented as mean ± SD. Differences between multiple groups were analyzed using one-way analysis of variance (ANOVA) with Dunnett’s post-test. Differences between two groups were analyzed using Student’s *t*-test. Statistical significance was set at *P* < 0.05.

## Results

### The Serum Nonylphenol Level Was Associated With Colorectal Cancer

**FIGURE 1:**
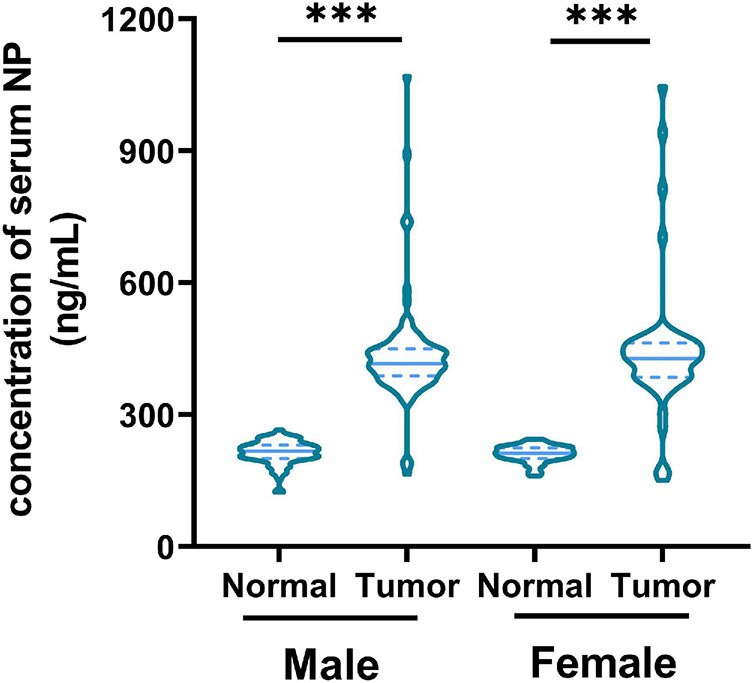
Concentration of nonylphenol (NP) in serum from healthy patients (*n* = 143) and patients with colorectal cancer (CRC; *n* = 143). ^∗∗∗^*p* < 0.001.

Serum NP levels in healthy patients (*n* = 143) and patients with CRC (*n* = 143) were tested. We found a higher concentration of serum NP in patients with CRC than in healthy individuals in both men and women (Figure 1). This result suggests that the concentration of serum NP is associated with CRC occurrence.

### Nonylphenol Exposure Induced Colorectal Cancer Cell Proliferation and Transcriptome Changes

**FIGURE 2:**
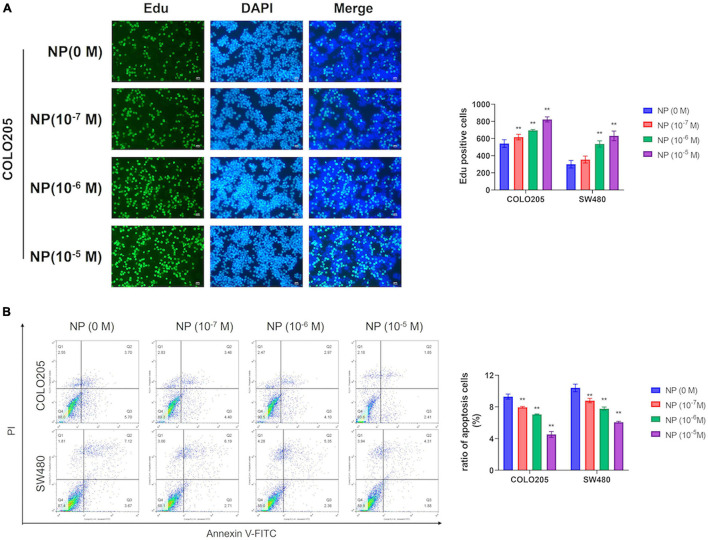
Nonylphenol (NP) induces colorectal cancer (CRC) cell proliferation and inhibits cell apoptosis. **(A)** COLO205 and SW480 cells treated with different concentrations of NP (0, 10^–7^, 10^–6^, and 10^–5^ M) for 24 h and tested by 5-ethynyl-2′-deoxyuridine (EdU) assay. Bar = 100 μm. Positive cells were measured, and the results are shown as bar charts. **(B)** NP treated COLO205 and SW480 cells stained with Annexin V-FITC/PI; cell apoptosis tested using flow cytometry. **p < 0.01.

**FIGURE 3:**
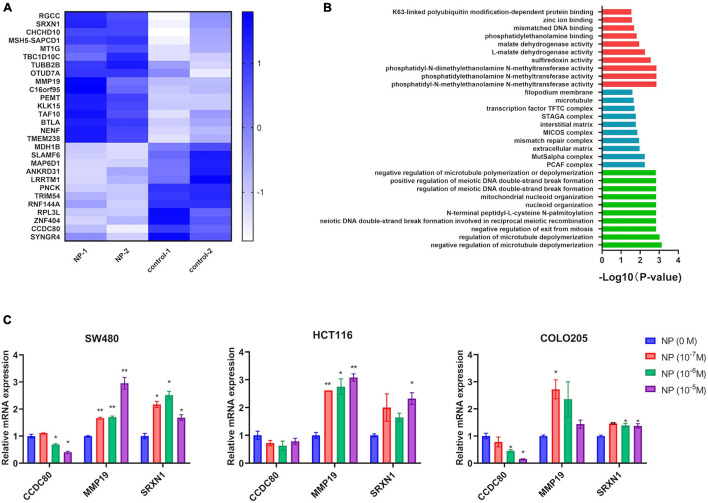
Nonylphenol (NP) influences the gene expression profile in colorectal cancer (CRC) cells. **(A)** Heatmap showing the differentially expressed genes in COLO205. **(B)** Gene ontology (GO) enrichment analysis of differentially expressed genes. Red: top 10 of molecular function terms; blue: top 10 of cellular component terms; green: top 10 of biological process terms. **(C)** RNA expression of CCDC8, MMP19, and SRXN1 in NP treated COLO205, SW480, and HCT116 cells as tested using quantitative PCR (qPCR). *p < 0.05, **p < 0.01, compared to NP (0 M).

We used different concentrations of NP (0, 10^–7^, 10^–6^, and 10^–5^ M) in treating COLO205 and SW480 cells. The Edu assay suggested that NP-induced CRC cell proliferation in a dose-dependent manner (Figure 2A and Supplementary Figure 2A). Furthermore, flow cytometry showed that NP inhibited apoptosis in a dose-dependent manner in CRC cells (Figure 2B). To explore the mechanism of NP on CRC cell progression, we performed transcriptome sequencing to analyze the transcript changes after NP exposure in CRC cells. We found 16 upregulated genes and 12 downregulated genes in COLO205 cells after NP treatment (Figure 3A). Gene ontology (GO) enrichment analysis suggested that NP treatment significantly influenced the processes associated with the extracellular matrix and cell cycle (Figure 3B). Quantitative PCR (qPCR) was performed to validate the sequencing results. The results showed that CCDC80 was downregulated and MMP19 and SRXN1 were upregulated in HCT116, SW480, and COLO205 cells after treatment with different concentrations of NP (Figure 3C).

### Expression of Coiled-Coil Domain Containing 80 Inhibited by Nonylphenol Treatment Was Associated With Colorectal Cancer

**FIGURE 4:**
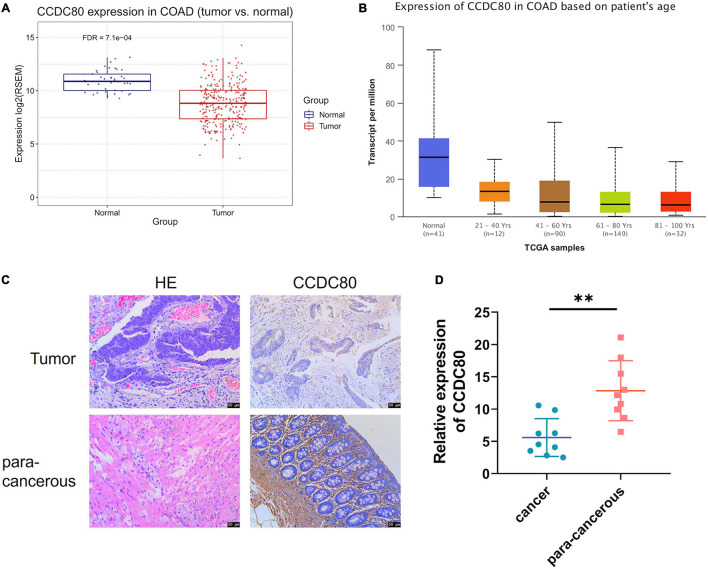
Expression of coiled-coil domain containing 80 (CCDC80) decreases in colorectal cancer (CRC) tissues. **(A)** CCDC80 expressed in CRC and normal tissues. **(B)** CCDC80 expression in CRC in patients with different age stages. **(C)** The expression of CCDC80 in CRC and para-cancerous tissues as detected by hematoxylin-eosin (HE) and immunohistochemistry (IHC) strain. Bar = 50 μm. **(D)** The positive expression of CCDC80 in CRC and para-cancerous tissues by IHC strain analyzed using the ImageJ software. ^∗∗^*p* < 0.01.

After TCGA analysis for the differentially expressed genes, we found that the expression of CCDC80, a gene downregulated by NP treatment, was significantly lower in tumor tissues than in normal tissues (Figures 4A,B). The expression of CCDC80 in CRC tissues and para-cancerous tissues confirmed the results of the TCGA analysis (Figures 4C,D).

### Nonylphenol-Induced Colorectal Cancer Cell Growth by Inhibiting Coiled-Coil Domain Containing 80 Expression

**FIGURE 5:**
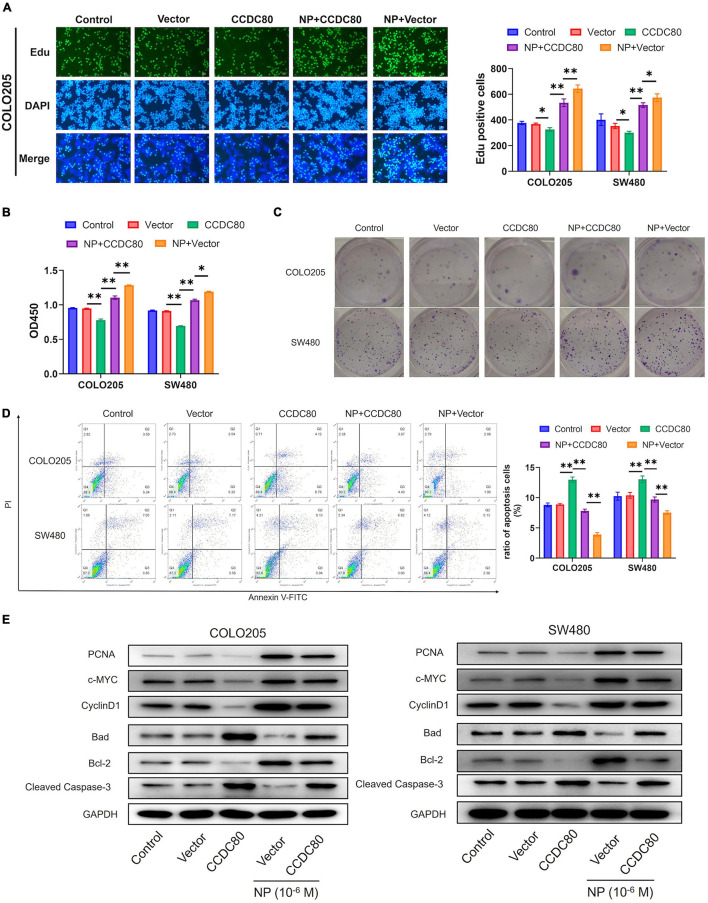
Overexpression of coiled-coil domain containing 80 (CCDC80) suppresses nonylphenol (NP)-induced colorectal cancer (CRC) cell growth. **(A)** Infected COLO205 and SW480 cells treated with NP (10^–6^ M) for 24 h and tested using EdU assay. Bar = 100 μm. Positive cells were measured, and the results are shown as bar charts. **(B)** The cell proliferation ability as tested using CCK8 assay in treated COLO205 and SW480 cells. **(C)** Colony forming assay performed to test the growth of treated cells. **(D)** Cell apoptosis in treated cells analyzed using flow cytometry. **(E)** The expression of cell proliferation-related proteins [proliferating cell nuclear antigen (PCNA), c-MYC, and CyclinD1] and cell apoptosis-related proteins (Bcl-2, Bad, and Cleaved Caspase-3) in treated cells analyzed using western blot assay. ^∗^*p* < 0.05, ^∗∗^*p* < 0.01.

To explore the roles of CCDC80 in NP-induced CRC cell growth, we constructed CCDC80 overexpressed stable infected CRC cell lines and then treated them with NP. EdU and CCK-8 analyses showed that overexpression of CCDC80 significantly suppressed CRC cell proliferation, even after NP treatment (Figures 5A,B and Supplementary Figure 2B). The results of the colony forming assay showed that CRC cell growth was significantly enhanced after NP treatment and suppressed by CCDC80 overexpression (Figure 5C). CCDC80 overexpression also significantly reduced NP-induced cell growth. Flow cytometry showed that overexpression of CCDC80 significantly induced cell apoptosis, even after NP treatment (Figure 5D). Western blot assay showed that, while NP treatment could induce the expression of proliferation-related proteins PCNA, c-MYC, and CyclinD1, CCDC80 overexpression could reduce it (Figure 5E). Furthermore, the expression of proapoptotic proteins (Bad and cleaved Caspase-3) was significantly decreased, and the expression of anti-apoptotic protein (Bcl-2) was significantly increased by NP treatment. Conversely, the overexpression of CCDC80 increased the expression of pro-apoptotic proteins and decreased the expression of anti-apoptotic proteins (Figure 5E and Supplementary Figures 3A,B). The overexpression of CCDC80 attenuated the effect of NP treatment on the expression of proliferation-related and apoptosis-related proteins.

### Coiled-Coil Domain Containing 80 Inhibited the Nonylphenol-Mediated ERK1/2 Activation

**FIGURE 6:**
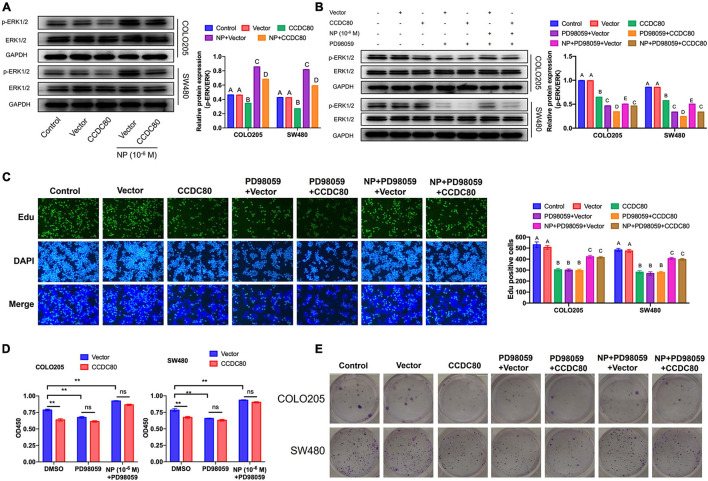
Coiled-coil domain containing 80 (CCDC80) suppresses colorectal cancer (CRC) cell proliferation by inhibiting ERK1/2 activity. **(A)** Infected COLO205 and SW480 cells were treated with nonylphenol (NP) (10^–6^ M) for 24 h. The expression of ERK1/2 and phosphorylated ERK1/2 (p-ERK1/2) in these treated CRC cells as analyzed using western blot assay. **(B)** Infected COLO205 and SW480 cells were treated by NP (10^–6^ M) or extracellular regulated protein kinases (ERK) inhibitor PD98059 for 24 h. The expression of ERK1/2 and phosphorylated ERK1/2 (p-ERK1/2) in these cells as analyzed using western blot assay. **(C)** Treated COLO205 and SW480 cells analyzed using EdU assay. Bar = 100 μm. Positive cells were measured, and the results are shown as bar charts. **(D)** The ability of cell proliferation tested using CCK8 assay in treated COLO205 and SW480 cells. **p < 0.01. **(E)** Colony forming assay performed to test the growth of treated cells.

**FIGURE 7:**
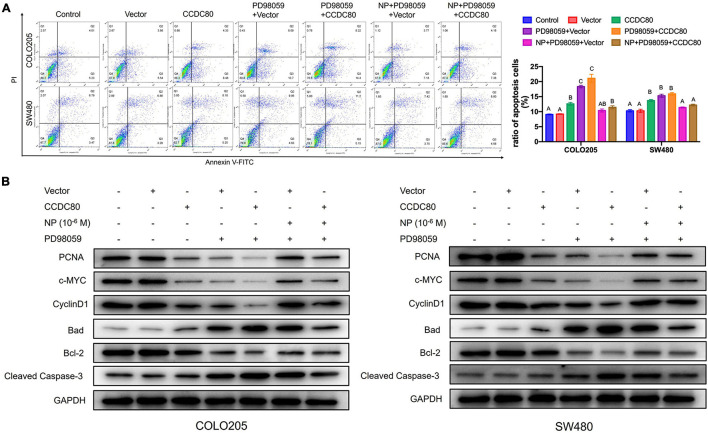
Coiled-coil domain containing 80 (CCDC80) increases colorectal cancer (CRC) cell apoptosis by inhibiting ERK1/2 activity. **(A)** Cell apoptosis in treated cells analyzed using flow cytometry. **(B)** The expression of cell proliferation-related proteins (PCNA, c-MYC, and CyclinD1) and cell apoptosis-related proteins (Bcl-2, Bad, and Cleaved Caspase-3) in treated cells analyzed using western blot assay.

Our previous study revealed that NP activated ERK1/2 (Xie et al., 2019). Therefore, we explored the relationship between the NP treatment, expression of CCDC80, and activation of ERK1/2. Western blot assay showed a decreased expression of phosphorylated ERK1/2, but its expression did not significantly change with the overexpression of CCDC80 after NP treatment (Figure 6A). We further investigated the effect of extracellular regulated protein kinases (ERK) inhibitor (PD98059) combined with NP in infected COLO205 and SW480 cells (Figure 6B). Treatment with the ERK inhibitor alone significantly inhibited the proliferation of CRC cells and induced apoptosis. The effect of the ERK inhibitor was attenuated by NP treatment but was not significantly enhanced by the overexpression of CCDC80 (Figures 6C–E, 7A). In addition, we performed western blot assay to analyze the expression of proliferation-related and apoptosis-related proteins and the activation of ERK1/2 after treatment with an ERK inhibitor combined with NP in infected CRC cells. Treatment with NP alone increased the expression of proliferation-related proteins (PCNA, c-MYC, and CyclinD1) and Bcl-2, while overexpression of CCDC80, especially when combined with an ERK inhibitor treatment, decreased the expression of these proteins. Furthermore, NP treatment induced the expression of anti-apoptotic proteins (Bad and cleaved Caspase-3), whereas this was reduced by the overexpression of CCDC80 combined with an ERK inhibitor (Figure 7B and Supplementary Figures 3C,D).

### Coiled-Coil Domain Containing 80 Inhibited Nonylphenol-Induced Tumor Growth *in vivo*

**FIGURE 8:**
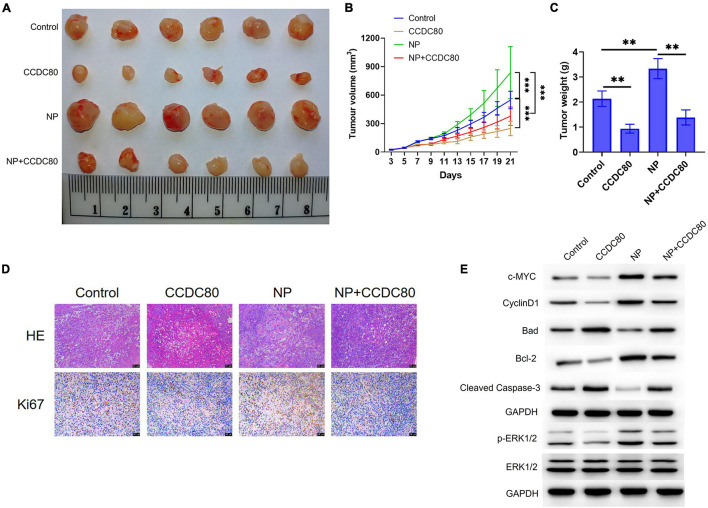
Overexpression of coiled-coil domain containing 80 (CCDC80) suppresses nonylphenol (NP)-induced tumor growth *in vivo*. **(A)** Photograph of tumor tissues after treatment. Bar = 50 μm. **(B)** The tumor volume measured every 2 days (*n* = 6). ^∗∗^*p* < 0.01, ^∗∗∗^*p* < 0.001. **(C)** The tumor weight measured after resection (*n* = 6). ^∗∗^*p* < 0.01. **(D)** The expression of Ki67 in tumor tissues as detected by hematoxylin-eosin (HE) and immunohistochemistry (IHC) strain. **(E)** The expression of cell proliferation-related proteins (PCNA, c-MYC, and CyclinD1), cell apoptosis-related proteins (Bcl-2, Bad, and Cleaved Caspase-3), ERK1/2, and p-ERK1/2 in tumor tissues analyzed using western blot assay.

A mouse xenograft model of human CRC cells was established to study the effect of CCDC80 on NP-induced tumor growth *in vivo*. The results showed that NP treatment significantly increased tumor growth, and that the overexpression of CCDC80 significantly suppressed tumor growth, even when co-treated with NP (Figures 8A–C and Supplementary Figure 4). IHC results showed lower expression of the proliferative marker Ki67 in the CCDC80 overexpression group (Figure 8D). Western blot assay showed similar effects of CCDC80 overexpression and NP treatment on the expression of proliferation-related proteins (PCNA, c-MYC, and CyclinD1), apoptosis-related proteins (Bcl-2, Bad, and cleaved Caspase-3), and activation of ERK1/2 in *in vitro* experiments (Figure 8E). These results revealed that CCDC80 inhibited NP-induced tumor growth.

## Discussion

The impact of environmental pollution on human health has aroused widespread concern. NP is an environmental hormone that has been shown to increase the risk of cancer. Recently, the relationship between NP exposure and incidence of CRC has been considered. A Spanish study also found a high risk of CRC in people living near industries releasing NP (Garcia-Perez et al., 2020). Because NP was wildly found in the environment and could be stored in human tissues, people in non-industrial areas may also be exposed to large amounts of NP. Our study also found that the serum NP concentration of patients with CRC was significantly higher than that of healthy individuals in both sexes. These results suggest that NP exposure increased incidence of CRC.

Many studies have suggested that NP could interact with estrogen receptors (ERs) and interfere with the estrogen signaling pathway to enhance the proliferation of ERs positive cells (Kang et al., 2012). Therefore, NP may promote estrogen-related cancer progression, such as breast and ovarian cancer (In et al., 2015; Kim et al., 2015). However, NP could also interact with the G protein-coupled ER involved in the non-classical ER pathway (Barton and Prossnitz, 2015). Different receptors may induce different estrogen-related cellular signaling (Marino et al., 2006; Filardo et al., 2007). Our previous study demonstrated that NP not only induced the expression of GPR30, but also interacted with it to enhance CRC cell proliferation (Xie et al., 2019).

In this study, we explored the mechanism of action of NP on CRC cell proliferation by RNA-sequencing. NP exposure could change the cellular response to the extracellular matrix and cell cycle signals. Furthermore, we found that CCDC80 was downregulated after NP treatment in three CRC cells and tissues. CCDC80, also known as downregulated by oncogenes 1 (DRO1), is shown to be downregulated in many types of cancers, including CRC, pancreatic cancer, and thyroid cancer (Visconti et al., 2003; Bommer et al., 2005). Loss of CCDC80 increased the development of carcinoma in ApcMin/− mice, suggesting that CCDC80 acts as a tumor suppressor in colon tumorigenesis (Grill et al., 2014). In our data, overexpression of CCDC80 significantly suppressed CRC cell proliferation and tumor growth and induced the expression of c-MYC. Meanwhile, the overexpression of CCDC80 significantly reduced NP-induced CRC cell proliferation. These results indicate that CCDC80 may be a potential target of NPs to induce CRC progression.

Previous studies suggested that the loss of CCDC80 could increase the phosphorylation of ERK (Grill et al., 2014; Gong et al., 2019), and that NP treatment activates the ERK1/2 pathway by interacting with GPR30 (Ge et al., 2014; Xie et al., 2019). Here, we found that the overexpression of CCDC80 reduced NP-induced ERK1/2 activity, and that an ERK inhibitor could eliminate the effect of CCDC80 overexpression on NP-mediated cell growth. These results indicate that the activation of ERK1/2 might be a key factor in CRC cell proliferation and that its activation is a result of NP-induced inhibition of CCDC80 expression. The ERK pathway regulates multiple cellular processes and participates in many tumor processes, including cell proliferation, apoptosis, invasion, and metastasis (Olea-Flores et al., 2019). These results indicate that CCDC80 suppresses NP-induced CRC cell growth by inhibiting the activation of ERK1/2. However, the mechanism by which NP treatment inhibits CCDC80 expression requires further study. Recent study suggested that activation of TGF-β signaling could inhibit the expression of CCDC80 (Hong et al., 2020), which suggested that NP might influence the expression of CCDC80 by activating other signaling pathways. Additionally, our data revealed that NP treatment activated ERK1/2, even when co-treated with CCDC80 overexpression. This might be because NP binds to GPR30 to activate the ERK pathway (He et al., 2009; Chakrabarti and Davidge, 2012; Bustos et al., 2017).

Previous studies suggested that natural NPs was a complex mixture which consisted with isomeric compounds that have different branched nonyl side chains (Guenther et al., 2006; Günther et al., 2017). Our study only analyzed the effect of 4-n-nonylphenol on the development of CRC, the different effect among the isomeric compounds needs more experiments to elucidate. Furthermore, why the serum NP content of CRC patients in the same area was higher than that of the normal population needs more further statistical analysis, such as different living habits, eating habits, and so on.

## Conclusion

In conclusion, our study confirmed the effects of NP exposure on cancer-related gene expression. The expression of CCDC80 were significantly downregulated by NP treatment. The overexpression of CCDC80 reduced the stimulatory effect of NP on the growth of CRC cells by inhibiting the NP-induced activation of ERK1/2. These findings provide insights on the oncogenic role of NPs and highlight the potential effect of CCDC80 in CRC.

## Data Availability Statement

The datasets presented in this study can be found in online repositories. The names of the repository/repositories and accession number(s) can be found below: RNA sequencing data to GEO database, the series number is GSE182226. The link is https://www.ncbi.nlm.nih.gov/geo/query/acc.cgi?acc=GSE182226.

## Ethics Statement

The studies involving human participants were reviewed and approved by the Medical Ethics Committee of the Zunyi Medical University [(2019) H-008]. The patients/participants provided their written informed consent to participate in this study. The animal study was reviewed and approved by the Animal Experiment Ethics Committee of the Zunyi Medical University [(2019) A-004].

## Author Contributions

JW and X-FY: conceptualization. JW, Y-WZ, N-JZ, SY, and D-JR: methodology. JW and Y-WZ: software. Y-WZ and N-JZ: validation. N-JZ: formal analysis and investigation. SY: resources. NH: data curation. JW: writing—original draft preparation. XC: writing—review and editing and visualization. X-FY: supervision and project administration. All authors agreed to be accountable for the content of the work.

## Conflict of Interest

The authors declare that the research was conducted in the absence of any commercial or financial relationships that could be construed as a potential conflict of interest.

## Publisher’s Note

All claims expressed in this article are solely those of the authors and do not necessarily represent those of their affiliated organizations, or those of the publisher, the editors and the reviewers. Any product that may be evaluated in this article, or claim that may be made by its manufacturer, is not guaranteed or endorsed by the publisher.
