# Supplementary material for: Coiled-Coil Domain Containing 80 Suppresses Nonylphenol-Induced Colorectal Cancer Cell Proliferation by Inhibiting the Activation of ERK1/2
Source: Front Cell Dev Biol. 2021 Oct 22;9:759820. doi: 10.3389/fcell.2021.759820 (PMC8570822; doi:10.3389/fcell.2021.759820)
Supplement: Supplementary file 1 [file Data_Sheet_1.DOCX]

Supplementary Material


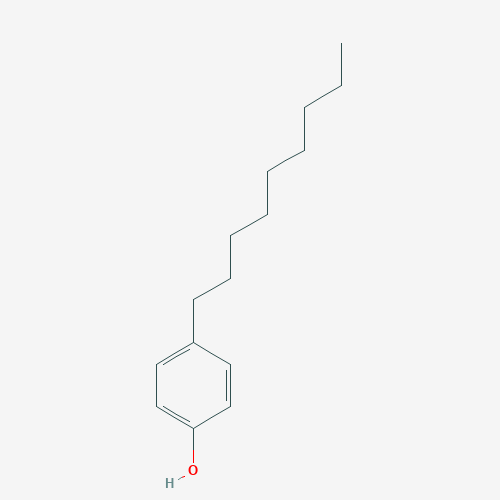


**Supplementary Figure 1.** The chemical structure of NP.


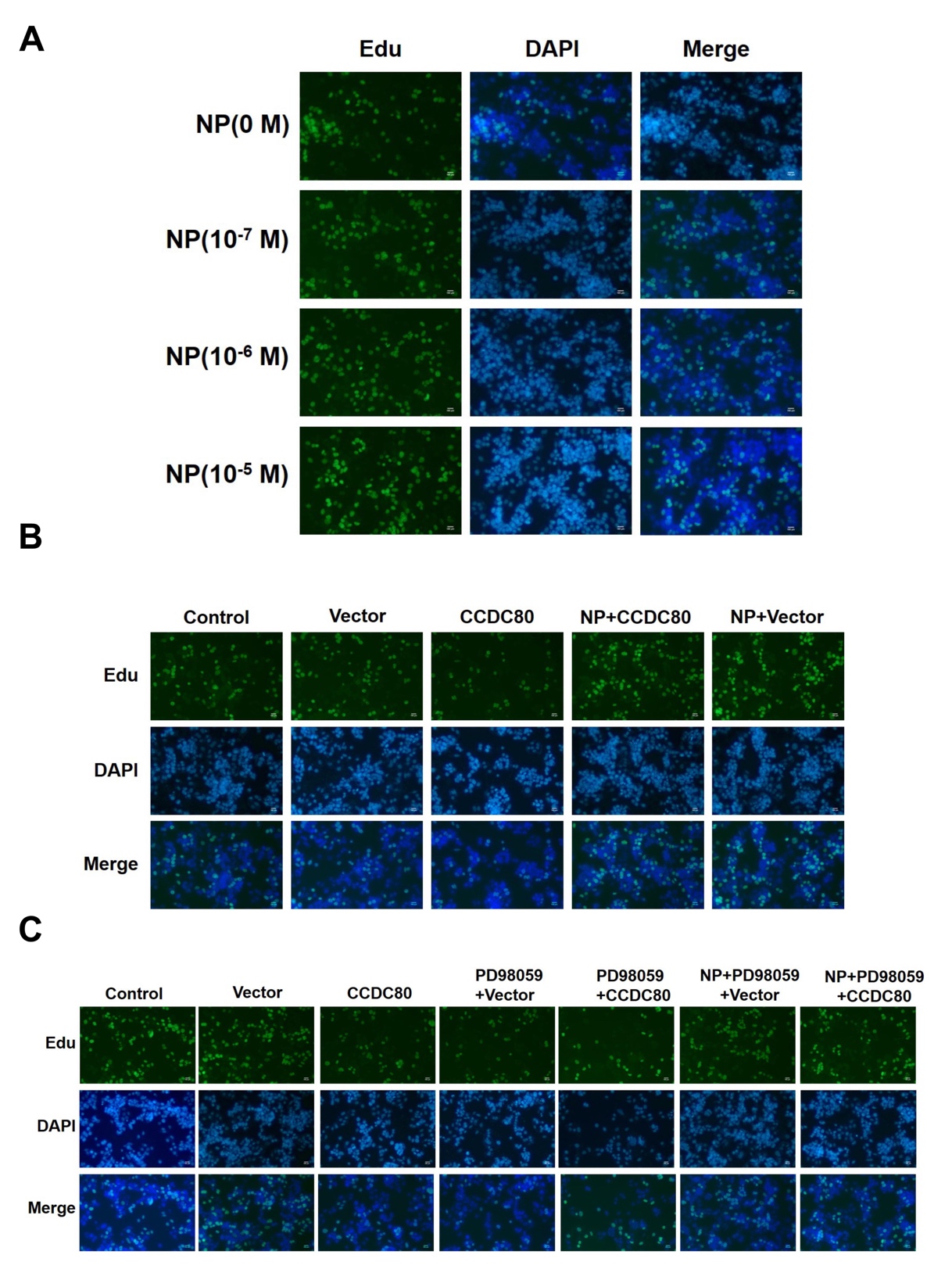


**Supplementary Figure 2.** Fluorescence microscope image of EdU assay of treated SW480 cells. Bar=100 μm.


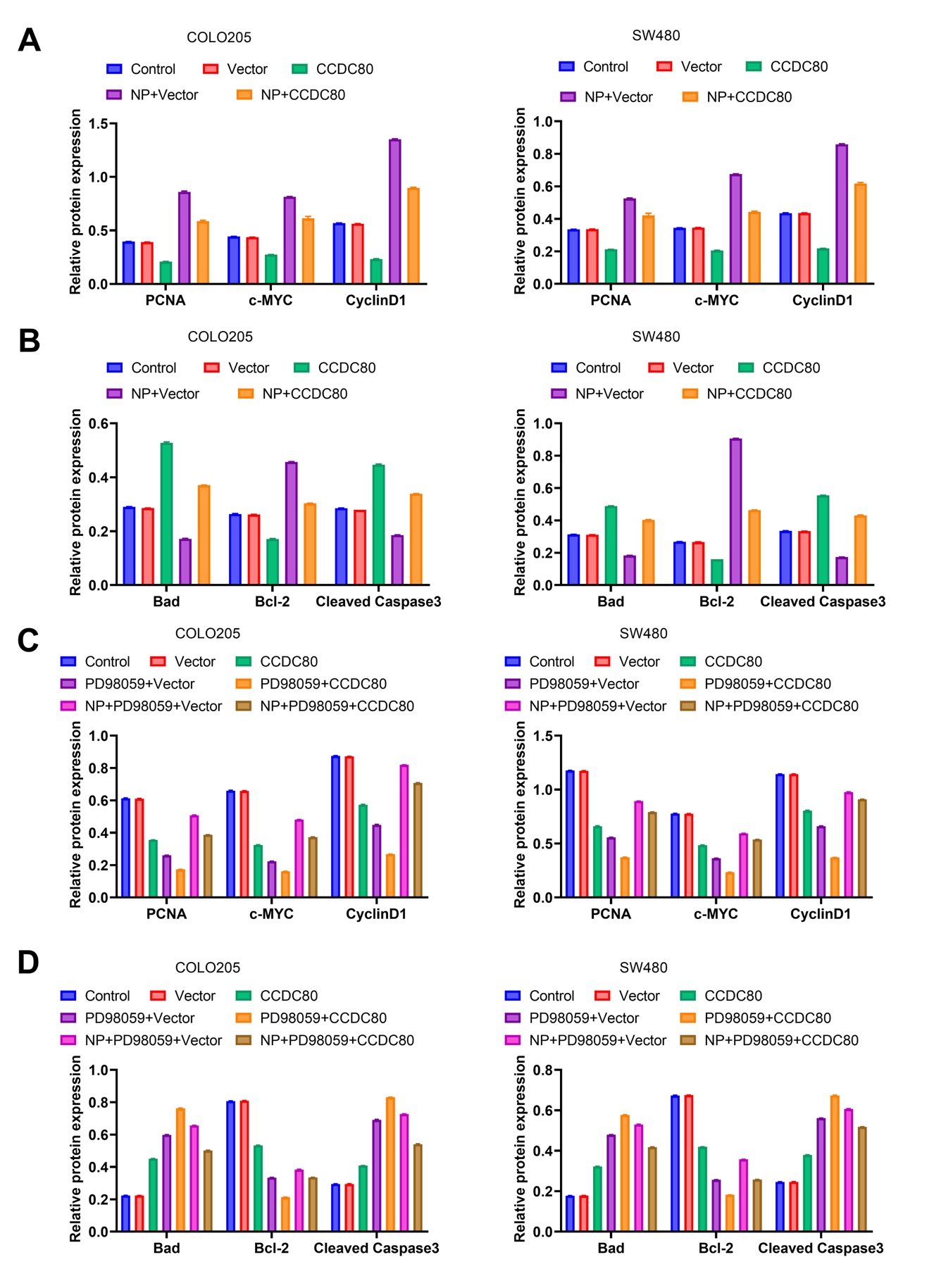


**Supplementary Figure 3.** Densitometry analysis of western blots.


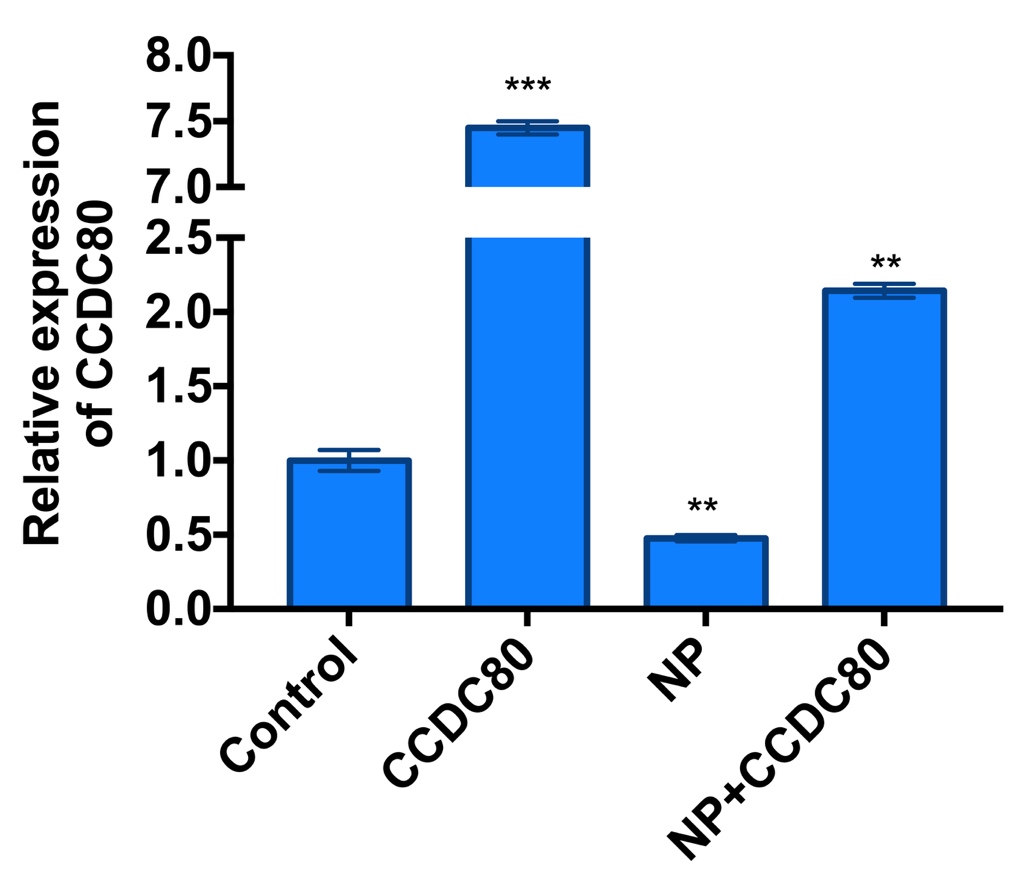


**Supplementary Figure 4.** The expression of CCDC80 in tumor after treatment. ***，p<0.001; **, p<0.01.


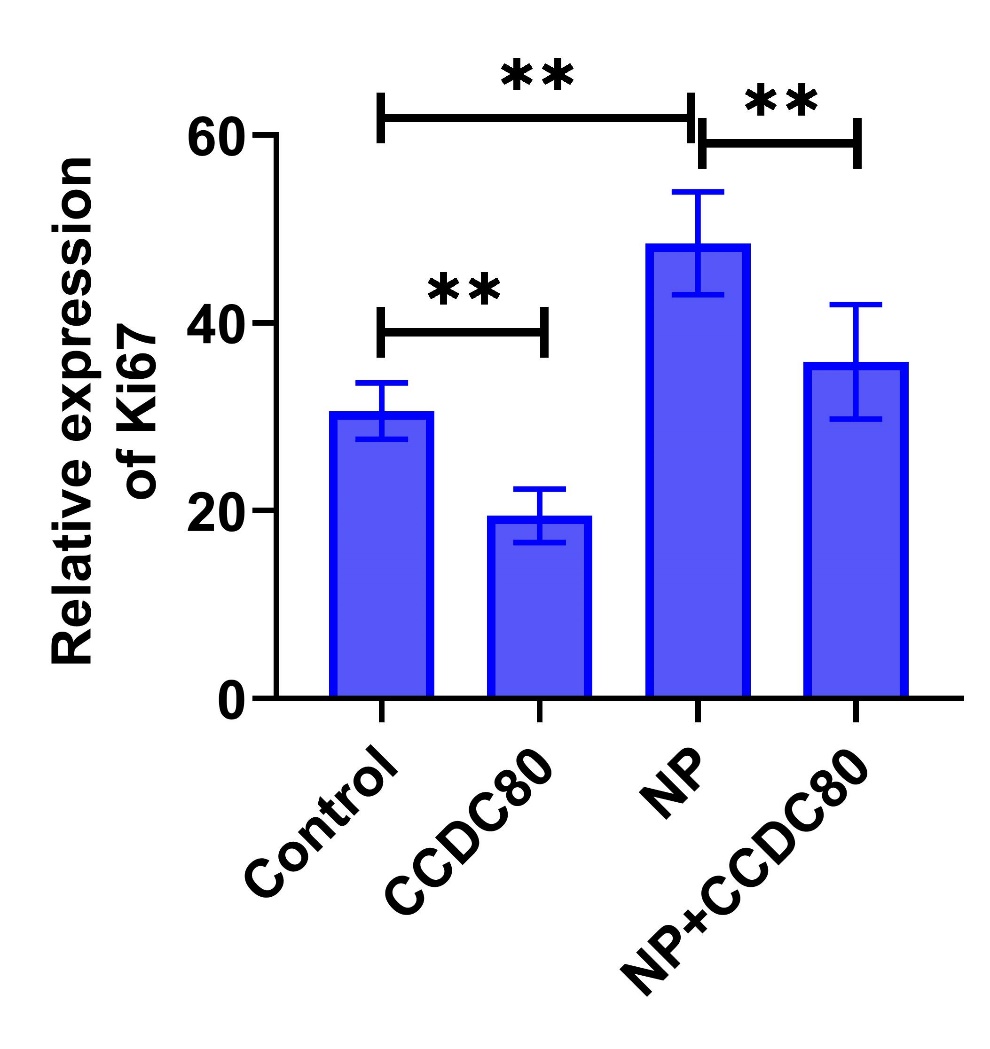


**Supplementary Figure 5.** The expression of Ki67 in tumor after treatment. **, p<0.01.
